# Tamoxifen Is Effective in the Treatment of *Leishmania amazonensis* Infections in Mice

**DOI:** 10.1371/journal.pntd.0000249

**Published:** 2008-06-11

**Authors:** Danilo C. Miguel, Jenicer K. U. Yokoyama-Yasunaka, Silvia R. B. Uliana

**Affiliations:** Departamento de Parasitologia, Instituto de Ciências Biomédicas, Universidade de São Paulo, São Paulo, Brazil; Hebrew University, Israel

## Abstract

**Background:**

Chemotherapy is still a critical issue in the management of leishmaniasis. Until recently, pentavalent antimonials, amphotericin B or pentamidine compounded the classical arsenal of treatment. All these drugs are toxic and have to be administered by the parenteral route. Tamoxifen has been used as an antiestrogen in the treatment and prevention of breast cancer for many years. Its safety and pharmacological profiles are well established in humans. We have shown that tamoxifen is active as an antileishmanial compound in vitro, and in this paper we analyzed the efficacy of tamoxifen for the treatment of mice infected with *Leishmania amazonensis*, an etiological agent of localized cutaneous leishmaniasis and the main cause of diffuse cutaneous leishmaniasis in South America.

**Methodology/Principal Findings:**

BALB/c mice were infected with *L. amazonensis* promastigotes. Five weeks post-infection, treatment with 15 daily intraperitoneal injections of 20 mg/kg tamoxifen was administered. Lesion and ulcer sizes were recorded and parasite burden quantified by limiting dilution. A significant decrease in lesion size and ulcer development was noted in mice treated with tamoxifen as compared to control untreated animals. Parasite burden in the inoculation site at the end of treatment was reduced from 10^8.5±0.7^ in control untreated animals to 10^5.0±0.0^ in tamoxifen-treated mice. Parasite load was also reduced in the draining lymph nodes. The reduction in parasite number was sustained: 6 weeks after the end of treatment, 10^15.5±0.5^ parasites were quantified from untreated animals, as opposed to 10^5.1±0.1^ parasites detected in treated mice.

**Conclusions/Significance:**

Treatment of BALB/c mice infected with *L. amazonensis* for 15 days with tamoxifen resulted in significant decrease in lesion size and parasite burden. BALB/c mice infected with *L. amazonensis* represents a model of extreme susceptibility, and the striking and sustained reduction in the number of parasites in treated animals supports the proposal of further testing of this drug in other models of leishmaniasis.

## Author Summary

Leishmaniasis is an antropozoonotic disease with a wide range of clinical manifestations. In humans, signs of disease vary from skin and mucosal ulcers to enlargement of internal organs such as the liver and spleen. The unicellular parasite *Leishmania amazonensis* is able to infect humans and cause localized or diffuse skin lesions. The treatment for this disease is difficult, as it requires prolonged and painful applications of toxic drugs that are poorly tolerated. Therefore, a key area in leishmaniasis research is the study of new therapeutic schemes and less toxic drugs. The present report is based on the investigation of tamoxifen's activity (a compound that has been in clinical use since the 1970s for the treatment of breast cancer) in the treatment of mice experimentally infected with *L. amazonensis*. We observed that infected mice treated with 20 mg/kg/day of tamoxifen for 15 days showed a significant clinical and parasitological response, with reduction in the size of lesions and ulcers and decreased numbers of parasites. These promising results pave the way for further testing of this drug as a new alternative in the chemotherapy of leishmaniasis.

## Introduction

Protozoan parasites of *Leishmania* genus are the etiological agents of leishmaniasis, a disease distributed worldwide with a broad spectrum of clinical manifestations according to the causative species and immunological status of the host. Leishmaniasis current therapy is mainly based on the systemic administration of toxic pentavalent antimonials or amphotericin B, drugs with several side effects, such as arrhythmia, nephro- and hepatotoxicity. Additionally, emergence of *Leishmania* strains resistant to antimonials has been reported [1],[2]. Recently, miltefosine has been approved in India for the therapy of visceral leishmaniasis [3], but its efficacy on the treatment of American cutaneous leishmaniasis has been shown to be variable depending on the causative species [4],[5],[6],[7]. Therefore, new alternatives for the treatment of leishmaniasis are greatly needed.

In South America, *Leishmania amazonensis* is one of the causative agents of localized cutaneous leishmaniasis and the most important agent of diffuse cutaneous leishmaniasis (DCL), a devastating disease with uncontrolled progression, characterized by multiple skin lesions and vaste numbers of amastigotes. As a rule, there is no satisfactory response to DCL treatment [8],[9].

We have previously shown that the antiestrogen tamoxifen, a drug extensively used as a chemotherapeutic and chemopreventive agent against breast cancer, presents leishmanicidal activity in vitro. This drug has a direct leishmanicidal effect and it also shifts the pH of parasitophorous vacuoles from acid to neutral, which in turn heightens the drug effect on amastigotes. Tamoxifen concentrations of approximately 10 µM inhibit 50% of *L. amazonensis* viability and growth in vitro [10]. In the present study we demonstrate that *L. amazonensis*-infected BALB/c mice treated with tamoxifen for 2 weeks presented a significant reduction in lesion size and parasite burden.

## Materials and Methods

The Ethics Committee that has approved this study is the Ethics Committee for Animal Experimentation of the Instituto de Ciências Biomédicas, University of São Paulo.

*L. amazonensis* promastigotes (MHOM/BR/1973/M2269) were grown in Medium 199 (Sigma-Aldrich) supplemented with 10% heat-inactivated fetal calf serum (FCS; Invitrogen) and incubated at 25°C. Female BALB/c mice (4–5 week-old) were inoculated with 5×10^6^ stationary-phase parasites at the base of the tail. Five weeks after infection, mice were randomly assigned into experimental groups (n = 7–10). Treated groups received intraperitoneal injections of 30.4 mg tamoxifen citrate/kg/day (the drug equivalent to 20 mg/kg/day tamoxifen) or 20 mg/kg/day meglumine antimoniate (Glucantime) for 15 days. Tamoxifen citrate was purchased from Sigma-Aldrich, USA; Glucantime was a kind gift from Sanofi-Aventis. Stock solutions of tamoxifen were prepared in saline every two days and stored at 4°C. Disease progression was evaluated once a week by recording the average diameter of the tail measured as the mean of tail base diameters in horizontal and vertical directions) and the ulcer size, expressed as the ulcer area in mm^2^. Measurements were taken with a caliper (Mitutoyo Corp., Japan). Body and uterus weights were also registered. Animal experiments were repeated four times and were approved by the Ethical Committee.

Parasite burden from infected tissue was quantified as described previously [11]. Promastigotes differentiated from lesion amastigotes were used on drug sensitivity assays while in the first passage in vitro. Cellular viability was assessed by measuring the cleavage of 3-(4,5-dimethylthiazol-2-yl)-2,5-diphenyl tetrazolium bromide (MTT; Sigma-Aldrich) by metabolically active cells as described [12].

Data on lesion progression were analyzed for statistical significance by using the non-parametric Mann-Whitney test (GraphPad Prism 5 software). Results of limiting dilution assay were analyzed based on two-tailed Student *t* test for paired samples using the ELIDA software. A result was considered significant at *P*<0.05.

## Results

**Figure 1:**
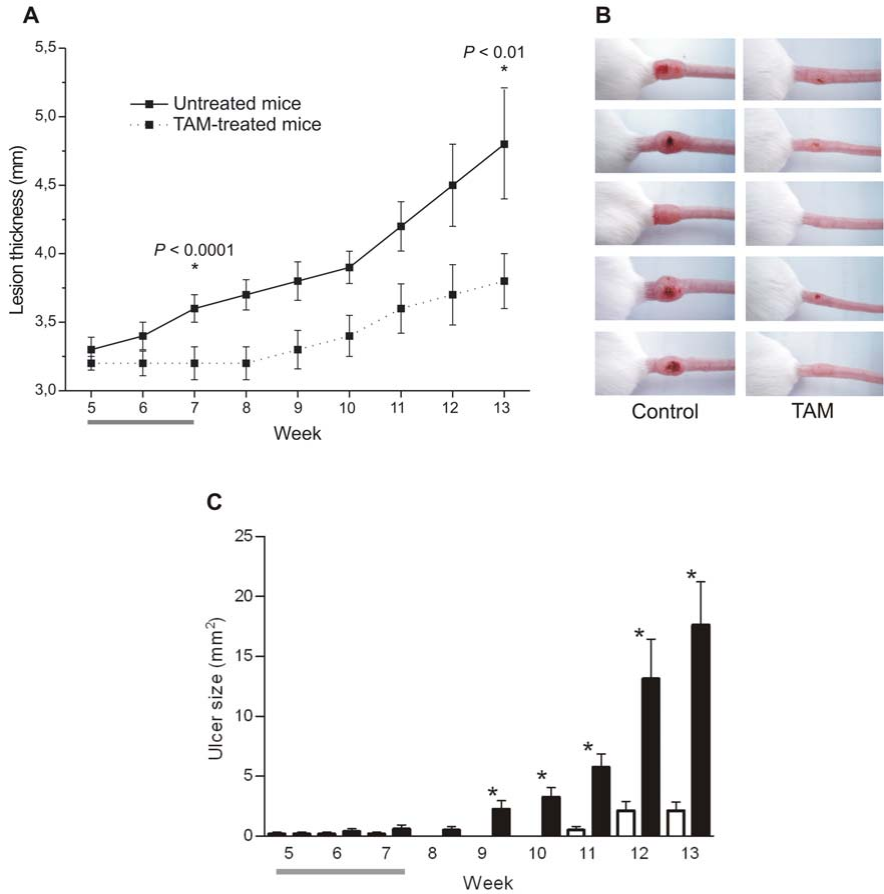
Follow up of *L. amazonensis* infection in BALB/c mice treated with tamoxifen (TAM). A: Progression of lesion thickness (mean±SD) in untreated (solid line) or TAM-treated (dotted line) mice. B: Macroscopical evaluation of lesions in untreated (left column) and TAM-treated mice (right column) at week 13 post-infection. C: Mean and SEM of ulcer size recorded from untreated (black bars) and TAM-treated animals (white bars). * *P*<0.005. Data represents one of three independent experiments. A and C: Horizontal grey bars indicate period of TAM administration (n = 10 per group from week 5 to 7 or n = 8 per group after week 7).

The treatment of *L. amazonensis-*infected BALB/c mice was initiated 5 weeks post-infection, time when lesions were already established and apparent. Mice were treated with 20 mg/kg/day tamoxifen intraperitoneally for 15 days. No toxic effects were detected during or after drug treatment. At the end of treatment, the average body weight in animals treated with tamoxifen was equivalent to values for the control group (untreated mice: 26.2±1.1 g; treated mice: 25.4±1.8 g) and the average weight of uteri indicated no significant alteration between tamoxifen-treated (0.18±0.4 g) and untreated mice (0.21±0.6 g). Figure 1A shows the progression of lesion size in untreated *versus* tamoxifen-treated mice. During and after tamoxifen administration we observed that treated animals presented less swelling at the infection site when compared to control animals. A statistically significant difference between the average thickness of lesions of untreated and tamoxifen-treated mice was evident on completion of treatment, at week 7 post-infection (*P*<0.001) and remained clear until the end of the experiment (week 13, *P*<0.01), when control mice had to be euthanized. Macroscopical aspects of the lesions are displayed in Figure 1B for untreated (left column) and tamoxifen-treated animals (right column).

Since *L. amazonensis* infection in BALB/c mice normally evolves from swelling at the infection site to an ulcerated lesion with loss of tissue, the measurement of lesion thickness can be misleading at late time-points. So, another criteria used for evaluating disease progression was the appearance and enlargement of ulcers. Tamoxifen treated mice showed a very significant delay in the development of ulcers when compared with untreated mice (Figure 1C).

**Figure 2:**
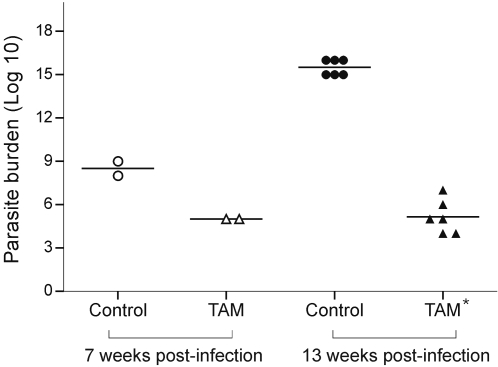
Parasite burden after treatment with tamoxifen (TAM). Number of parasites recovered by limiting dilution from mice infected with *L. amazonensis* and treated with TAM (triangles) and untreated controls (circles). Parasites were quantified from lesions removed immediately after the interruption of treatment (7 weeks post-infection; empty symbols; n = 2) or 6 weeks after interruption of treatment (13 weeks post-infection; filled symbols; n = 6). * *P<*0.0001.

Parasite burden in tamoxifen-treated animals was evaluated immediately after the interruption of treatment (7 weeks after infection) and 6 weeks later (13 weeks after infection). As shown in Figure 2, a significant decrease on total parasite numbers per lesion on tamoxifen-treated animals was observed in both time points. At the end of the experiment, the average number of parasites was reduced by at least 99.7% in treated groups, as compared to untreated animals. These results were reproduced in 3 independent experiments.

In order to evaluate the activity of tamoxifen in parallel with a standard drug, a fourth experiment was performed with groups of 10 mice treated with 20 mg/kg/day tamoxifen, 20 mg/kg/day Glucantime or mock-treated with saline. Treatment was initiated 4 weeks after infection and carried on for 15 days with daily intraperitoneal infections. As shown in Table 1, three weeks after the end of treatment, there was no difference in the average size of lesions between mock and Glucantime-treated mice. The group that received tamoxifen showed a significant decrease in lesion thickness. Parasite burden was determined for the lesion site, draining lymph node and spleen. There was a significant reduction in the numbers of parasites recovered from tamoxifen treated mice as compared to mock or Glucantime groups both at the lesion and lymph node. No parasites were recovered from the spleen in any of the groups (Table 1). Therefore, tamoxifen proved to be more effective in this experimental model than the standard drug.

**Table 1:** Evaluation of disease progression and parasite burden in *L. amazonensis*-infected mice 3 weeks after the end of treatment.

		Saline	Glucantime (20 mg/kg/d) a	Tamoxifen (20 mg/kg/d)
**Lesion thickness (mm) ** b		4.0±0.2	4.1±0.2	3.6±0.1
**Parasite burden**	**Lesion site ** c	10^5.4±0.7^	10^8.3±0.8^	10^3.0±0.1^
	**Lymph node ** d	10^3.7±0.7^	10^5.3±1.2^	10^2.0±0.1^
	**Spleen**	nd^e^	nd	nd
**Spleen weight (g)**		0.1±0.0	0.1±0.0	0.1±0.0

a equivalent to 5.4 mg Sb^V^/kg/day.

b Saline *vs* Tamoxifen: *P*<0.05 and Glucantime *vs* Tamoxifen: *P*<0.005.

c, d Saline *vs* Tamoxifen and Glucantime *vs* Tamoxifen: *P*<0.05.

e (nd) not detectable.

Finally, we investigated whether parasites remaining in tamoxifen treated groups were less sensitive to the drug. MTT viability assays showed that tamoxifen's activity against promastigotes derived from parasites extracted from treated or untreated mice remained unchanged with EC 50% of 11.5±1.1 and 12.8±2.8 µM, respectively. Therefore, remaining parasites did not develop resistance to tamoxifen during treatment.

## Discussion

Our data reveal a significant effect of tamoxifen in the reduction of skin lesions caused by *L. amazonensis* in BALB/c mice. Effectiveness was apparent not only as reduced swelling and ulceration in treated animals but also as an important reduction in parasite burden.

The experimental model of infection used in this study is one of extreme susceptibility. BALB/c mice infected subcutaneously with *L. amazonensis* develop progressive swelling at the inoculation site, followed by ulceration and loss of tissue simultaneous with the appearance of methastasis at distant sites. The treatment did not lead to sterile cure of lesions but *Leishmania* parasites have been shown to remain present and viable, although in decreased numbers, after treatment with antimonials in a variety of animal models, as well as in humans. The lack of clinical or parasitological response to Glucantime in *L. amazonensis* BALB/c infected mice has been reported previously [13].

Furthermore, the timing for initiation of treatment can significantly influence the disease outcome, as stressed by previous studies [14]. Our experimental treatments were initiated 30–35 days after mice infection, an interval of time that allowed the establishment of disease and when infection sites were already swollen and, in some animals, had started ulcerating. So, the data shown here imply that the intraperitoneal administration of tamoxifen resulted in a remarkable response to treatment. We are currently evaluating tamoxifen's efficacy in the treatment of other models of cutaneous and visceral leishmaniasis. We have shown in vitro that tamoxifen leishmanicidal effect is independent of the estrogen receptor [10] and therefore it is unlikely that response to treatment would be different in male or female mice. Indeed, preliminary results obtained in *Leishmania chagasi* infected hamsters show no gender-related effect on the anti-leishmanial response to tamoxifen.

Apart from its direct leishmanicial activity, tamoxifen mode of action in vivo could involve other pathways favouring amelioration of the infection. Tamoxifen has been reported to increase synthesis of inducible nitric oxide synthase and production of nitric oxide [15]. We did not detect differences in the accumulation of nitrate on supernatants of *L. amazonensis* infected macrophages treated or not treated with tamoxifen (data not shown).

The metabolite profile of tamoxifen varies in different animal models [16]. This drug has been used in mice in a variety of doses and administration schemes. The dosage employed in this study was chosen based on previous reports showing that, in mice, daily intraperitoneal injections of 25 to 100 mg/kg of tamoxifen resulted in drug serum levels similar to those observed in patients [17].

This antiestrogen has been widely used for treatment and prevention of breast cancer [18]. The most serious side effect observed on clinical grounds is an increased risk for endometrial cancer which appears after prolonged use. Effects observed in our experiments suggest that antileishmanial therapy with tamoxifen would not require extensive periods of treatment. We did not detect changes in uterine weight in treated female mice, a well-established parameter for evaluation of tamoxifen's toxicity [19]. Tamoxifen administered at 40 mg/kg/day for 4 weeks has been recently shown to impair bone growth in rats [20] raising concerns on the application of this drug to treat leishmaniasis in children. We are also investigating the effect of other selective estrogen receptor modulators with different effects in bone metabolism, like raloxifene, as antileishmanial drugs.

The potential value of tamoxifen for treating human leishmaniasis needs further evaluation. To the best of our knowledge, this is the first report of an in vivo investigation on tamoxifen's efficacy against *Leishmania* infection and points to a new alternative in the treatment of leishmaniasis.
